# Effects of miR-214 on adenosine A2A receptor and carboxymethyl chitosan nanoparticles on the function of keloid fibroblasts and their mechanisms

**DOI:** 10.1038/s41598-024-54125-6

**Published:** 2024-02-28

**Authors:** Yong Du, Jing Liu, Qing Hao, Shun Wang, Aijun Zhang, Yongzhong Li, Ninghan Feng

**Affiliations:** 1 Department of Plastic Surgery, Jiangnan University Medical Center, Wuxi City, 214000 China; 2 Department of Plastic Surgery, Affiliated Hospital of Xuzhou Medical University, Xuzhou City, 221000 China; 3 Department of Urology, Jiangnan University Medical Center, Wuxi City, 214000 China; 4 Department of Plastic Surgery, NO.2 Wuxi People’s Hospital, Affiliated Wuxi Clinical College of Nantong University, Wuxi, 214000 China

**Keywords:** miR-214, Keloid fibroblasts, Adenosine A2AR, Cell apoptosis, Cell proliferation, Carboxymethyl chitosan nanoparticles, Cell biology, Chemical biology

## Abstract

This work prepared and investigated the impact of carboxymethyl chitosan nanoparticles (MC-NPs) on the proliferative capability of keloid fibroblasts (KFBs) while analyzing the mechanistic roles of miR-214 and adenosine A2A receptor (A2AR) in fibroblasts within hypertrophic scars. MC-NPs were synthesized through ion cross-linking, were characterized using transmission electron microscopy (TEM) and laser particle size scattering. The influence of MC-NPs on the proliferation capacity of KFBs was assessed using the MTT method. Changes in the expression levels of miR-214 and A2AR in KFBs, normal skin fibroblasts (NFBs), hypertrophic scar tissue, and normal skin tissue were analyzed. KFBs were categorized into anti-miR-214, anti-miR-NC, miR-214 mimics, miR-NC, si-A2AR, si-con, anti-miR-214+ si-con, and anti-miR-214+ si-A2AR groups. Bioinformatics target prediction was conducted to explore the interaction between miR-214 and A2AR. Real-time quantitative PCR and immunoblotting (WB) were employed to detect the expression levels of miR-214, A2AR, apoptotic protein Bax, and TGF-β in different cells. Cell counting kit-8 (CCK8) and flow cytometry were employed to assess cell proliferation activity and apoptosis. The results indicated that MC-NPs exhibited spherical particles with an average diameter of 236.47 ± 4.98 nm. The cell OD value in the MC-NPs group was lower than that in KFBs (*P* < 0.05). The mRNA levels of miR-214 in KFBs and hypertrophic scar tissue were lower than those in NFBs and normal tissue (*P* < 0.001), while the mRNA and protein levels of A2AR were significantly elevated (*P* < 0.05). Compared to the control group and anti-miR-NC, the anti-miR-214 group showed significantly increased cell OD values and Bcl-2 protein expression (*P* < 0.001), decreased levels of apoptotic gene Bax protein, TGF-β gene mRNA, and protein expression (*P* < 0.001). Continuous complementary binding sites were identified between miR-214 and A2AR. Compared to the control group, the si-A2AR group exhibited a significant decrease in A2AR gene mRNA and protein expression levels (*P* < 0.001), reduced cell viability (*P* < 0.001), increased apoptosis rate (*P* < 0.001), and a significant elevation in TGF-β protein expression (*P* < 0.001). miR-214 targetedly regulated the expression of A2AR, inducing changes in TGF-β content, promoting the proliferation of keloid fibroblasts, and inhibiting cell apoptosis.

## Introduction

Keloid is a common and relatively serious pathological scar, characterized by abnormal proliferation (*Pro*) of connective tissue after skin trauma. The main features include excessive fibroblast *Pro* and extracellular matrix deposition, clinically referred to as “benign tumors of the skin”. Keloids appear as “crab-like swelling”, adversely affecting the patient’s appearance and causing significant itching and pain, leading to serious impacts on the patient’s physical and mental health, organ dysfunction, and mobility^1^. Keloids grow beyond the boundaries of the original wound, but they do not spontaneously regress or disappear over time. The incidence of keloids shows considerable racial differences^2^, with higher rates in Asians and Blacks. In China, the incidence of keloids is as high as 3.4%^3^, with a higher incidence in young people and a marked familial predisposition^4^. Currently, there is no specific treatment for keloids, and clinical treatments mainly include surgery combined with radiation therapy or physical compression, radiation, and drug therapy, which are challenging and often ineffective. Some researchers believe that surgery combined with radiation therapy is an effective treatment for keloids, with early radiation therapy potentially reducing the recurrence rate^5^, but actual clinical results show that this treatment methodology is not very effective, and surgical treatment is prone to recurrence^6^. Radiation therapy often leads to acute or chronic toxic reactions^7^. Hence, further exploration of the pathogenesis of keloids is of positive significance for early intervention and preventive measures.

Nanomaterials have extensive applications in medicine and bioengineering^8,9^. Carboxymethyl chitosan nanoparticles (MC-NPs) are a type of nanomaterial with significant potential for diverse applications. Carboxymethyl chitosan is a product obtained through the reaction of chitosan with compounds like methacrylic acid, possessing excellent biocompatibility and biodegradability^10^. It can be processed into particulate form through nanotechnology, offering a larger surface area and unique electrostatic properties^11^. MC-NPs exhibit favorable biocompatibility and degradability, serving as carriers for drug delivery systems. They can encapsulate and protect drugs, facilitating targeted drug release^12^. Additionally, MC-NPs find applications in fields such as biosensors, drug delivery, and wound repair, demonstrating promising prospects as biomedical materials^13,14^. However, the current application of these nanoparticles in KFBs is still in its early research stages, with limited relevant studies conducted thus far.

The occurrence of scars is related to mechanisms such as injury inflammation, changes in growth factors, and the renewal of vascular endothelial growth factor^15^. Adenosine is an endogenous purine nucleoside that participates in the endogenous signal transduction process by activating and binding G protein-coupled receptors. The adenosine A2A receptor (A2AR) is highly expressed in monocytes-macrophages, neutrophils, and T and B lymphocytes. Current research results indicate that knocking down adenosine A2ARs in mice significantly reduces skin wound repair and healing ability^16^. Zheng et al.^17^ found that the local application of A2AR agonists can promote wound healing, and its healing effect is drastically superior to platelet-derived growth factor. Guerrero^18^ pointed out that adenosine A2ARs act in wound healing and scar formation. Hence, adenosine A2ARs play an important role in skin wound repair, healing, and scar formation processes. Nevertheless, the specific mechanism of adenosine A2ARs in scars is not yet clear and further research is needed. MicroRNAs (miRNAs) are associated with various biological processes such as the translation of target genes, individual organ development, cell *Pro*, differentiation, apoptosis (*Apo*), autophagy, and tumor occurrence. Currently, research results show that abnormal levels of miRNA 214 (miR-214) are correlated with inflammatory and immune responses^19^. Bioinformatics research results indicate that miR-214 may be involved in inflammation by regulating A2AR^20^.

In conclusion, the role of MC-NPs in KFBs warrants further investigation, and whether miR-214 is involved in the regulation of A2AR in the biological processes of KFBs remains to be elucidated. Therefore, in this work, MC-NPs were synthesized using an ion-crosslinking method, and their impact on the proliferation ability of keloid fibroblasts was analyzed. Simultaneously, this work explored the roles of miR-214 and A2AR in the biological behavior of KFBs at the molecular and cellular levels, aiming to provide a theoretical foundation for the study of the mechanisms underlying keloid formation and to offer new insights into targeted treatment strategies for keloids.

## Materials and methodologies

### Experimental material

Human keloid fibroblasts (KFBs) and normal human skin fibroblasts (NFBs) were purchased from the typical culture collection committee of the Chinese Academy of Sciences. Fetal bovine serum (FBS) and Dulbecco’s Modified Eagle Medium (DMEM) were purchased from Gibico in the United States. 0.25% trypsin + EDTA, phosphate-buffered saline (PBS), and gentamicin were from Sigma-Aldrich in the United States. Chitosan was sourced from Sigma Reagent Company. Analytical grade anhydrous ethanol, hydrochloric acid, glacial acetic acid, methanol, chloroacetic acid, anhydrous calcium chloride, and other reagents were purchased from China National Pharmaceutical Group Chemical Reagent Co., Ltd. Lipofectamine TM 2000 and TRIzol reagent were purchased from Invitrogen in the United States. Anti-miR-214 and its control (anti-miR-NC), miR-214 mimics and its control (miR-NC), and si-A2AR and its control (si-con) were purchased from Shanghai Jima Pharmaceutical Technology Co., Ltd. PCR primers were provided by Suzhou Genewiz Biotechnology Co., Ltd. M-MLV Reverse Transcriptase Kit was purchased from Dalian TaKaRa Bio Co., Ltd. The Annexin V-FITC/PI *Apo* detection kit, cell counting kit 8 (CCK-8), and horseradish peroxidase (HRP)-conjugated donkey anti-goat IgG were from Shanghai Beyotime Biotechnology Co., Ltd. The goat anti-A2AR monoclonal antibody was purchased from Everest Biotech Ltd. monoclonal antibody was purchased from Shanghai Kangcheng Co., Ltd. The HRP-conjugated goat anti-mouse IgG was from Beijing Zhongsan Jinqiao Biotechnology Co., Ltd. The magnetic stirrer was obtained from Gongyi Yingyu Yuhua Instrument Factory. The AVATAR-360 Fourier-transform infrared spectrometer was purchased from Nicolet Instrument Corporation, USA. The Bettersize2000 laser particle size analyzer was acquired from Shanghai Keqi Instrument Equipment Co., Ltd. The EM 400 transmission electron microscope was sourced from PHILIPS, Netherlands. The Real-time PCR instrument (7500 model) was purchased from ABI in the United States.

### Tissue origin of keloid

The scar tissue and normal skin tissue were collected from 50 patients who visited our hospital from January 2019 to December 2022. All individuals were above the age of 18 years old. Using aseptic techniques, the epidermis and subcutaneous tissue was removed as much as possible. All collected scar tissue was confirmed by the pathology department. The diagnostic criteria for scar tissue were as follows^21^: fibroblasts in the scar tissue exhibit a ruptured appearance, irregular arrangement, abundant mucoid interstitium, and more hyaluronic acid in the epidermis. The inclusion criteria for scar tissue are: (1) a disease course of more than nine months without spontaneous acute regression; (2) recurrence after surgical or other treatment; and (3) skin damage exceeding the initial injury site and invading surrounding skin. The exclusion criteria are: (1) patients who had received treatment with steroids, radiotherapy, or other drugs; (2) patients with other skin diseases; (3) patients with immune or infectious diseases; and (4) patients with tumors or hereditary diseases. Approval for this study’s content was granted by the Medical Ethics Committee of the Second People’s Hospital of Wuxi City. All methods strictly adhered to relevant guidelines and regulations. Both the research patients and their family members were informed about the study details, and written consent was obtained from all participants who were aware of the study's content and willingly agreed to participate in the research.

### Preparation of MC-NPs

The method utilized by Migone et al.^22^ served as a reference for the preparation of MC-NPs using an ion-crosslinking approach, which was further optimized. In a beaker, 10 g of chitosan, 90 mL of isopropanol, and 14.0 g of solid NaOH were added, followed by the addition of a specific amount of water to reach a total volume of 500 mL. The mixture was stirred and allowed to swell for 1 h at 50 °C. Subsequently, a solution of chloroacetic acid in isopropanol (W:V = 3:4) was slowly added at 60 °C, and the reaction was proceeded for 7 h. After cooling to room temperature, the mixture was filtered, and the residue was collected. This was followed by three cycles of impurity removal and dehydration using 70% ethanol and 95% ethanol solutions (2 h each time). The resulting carboxymethyl chitosan was obtained after freeze-drying. To prepare MC-NPs, CaCl_2_ was used as the ion-crosslinking agent. The specific procedure involved preparing a 0.5 mg/mL solution of carboxymethyl chitosan. Then, 5 mL of the carboxymethyl chitosan solution was mixed with 2 mL of the crosslinking agent solution at room temperature and stirred for 30 min. The precipitate was collected by centrifugation at 20,000 rpm, and after freeze-drying, the MC-NPs were obtained.

### Characterization performance of MC-NPs

Fourier-transform infrared spectroscopy (FTIR) analysis: the dried MC-NPs were thoroughly mixed with KBr, pressed into transparent pellets, and scanned in the range of 400–4000/cm using an infrared spectrometer.

Transmission electron microscopy (TEM) analysis: a certain amount of MC-NPs was dissolved in triple-distilled water to prepare a suspension. The suspension was then dropped onto a copper grid, air-dried at room temperature, and observed under a TEM at 80 kV acceleration to examine the morphology of MC-NPs.

Particle size distribution analysis: a specific amount of MC-NPs was dissolved in triple-distilled water to prepare a suspension. The suspension was subjected to laser particle size analysis using a laser diffraction particle size analyzer. The detection conditions were set as follows: detection angle of 90°, wavelength (λ) of 670 nm, and temperature of 25.2 °C.

### Cell culture and grouping

DMEM culture medium with 10%FBS was applied to KFBs and incubated in a cell culture incubator (Thermo Fisher Scientific) at 37 °C and 5%CO_2_. When the cell density reached 80–90%, passaging was performed. All cells were assigned into eight groups: anti-miR-214, anti-miR-NC, miR-214 mimics, miR-NC, si-A2AR, si-con, anti-miR-214+ si-con, and anti-miR-214+ si-A2AR. Simultaneously, untreated KFBs were used as a control group, and the impact of MC-NPs on the proliferation ability of KFBs was assessed using the MTT assay.

### Double luciferase reporter gene experiment and cell transfection

Wild-type containing the miR-214 binding site (WT-A2AR), mutant-type containing the mutated sequence of the miR-214 binding site (MUT-A2AR), anti-miR-214 (5′-ACTGCCTCTGCCTGCTGT-3′), anti-miR-NC (5′-CAGTACTTTTGTGTAGTACAA-3′), miR-214 mimics (5′-ACAG CAGGCACAGACAGGCAGU-3′), miR-NC (5′-CAGUACUUUGUGUAGUA CAA-3′), si-A2AR (5′-CUGUGGUCGAUCCUGCUG-3′), si-con, anti-miR-214+ si-con, and anti-miR-214+ si-A2AR luciferase reporter gene vectors were transfected using the lipofection methodology. For the transfection procedure, 2 × 10^5^ 293 T cells were seeded in complete medium 24 h before transfection. When the cell confluency reached 70%, Lipofectamine™ 2000 transfection reagent was used for transfection. Specifically, 50 μL of Opti-MEM was utilized to dilute plasmid DNA, which was then mixed and incubated with 2 μL of Lipofectamine™ 2000 at 25 °C for 5 min. The transfection reagent and plasmid DNA were then mixed and incubated at 25 °C for 20 min. The mixture (100 μL) was applied to 24-well plates containing KFBs medium (300 μL) without serum or antibiotics, and then followed by incubation at 37 °C and 5% CO_2_ for 6 h. Medium was then removed, and complete medium containing serum and antibiotics was supplemented. The cells were further incubated at 37 °C and 5%CO_2_ for 48 h. The luciferase activity was detected to screen for stable transfection cell lines.

### Gene mRNA level determined by RT fluorescence qPCR

The total RNA was extracted from the scar tissue, normal human skin tissue, NFBs, and various groups of KFBs using the TRIzol method. After testing the extracted total RNA by agarose gel electrophoresis, the M-MLV Reverse Transcriptase kit was employed for reverse transcription and cDNA synthesis. The reverse transcription program set at 37 °C for 15 min and 85 °C for 5 s. Internal reference genes, β-actin, and U6 were used. RT fluorescence quantitative PCR (qPCR) was conducted to analyze the levels of each gene in the cells. The reaction system consisted of 20 μL, including 10 μL 2 × SYBR® Premix Ex Taq II, 0.2 μL upstream and downstream primers each, 0.4 μL 50 × ROX Reference Dye II, 5 μL cDNA template, and 4.2 μL ultra-pure water. Each reaction was repeated 3 times, and Real-time PCR instrument was used for PCR amplification with an annealing temperature of 60 ℃ and annealing time of 30 s for 30 cycles. The relative level was calculated using the 2^−ΔΔct^ methodology. Primer sequences for different genes are listed in Table 1.

**Table 1:** PCR primer sequences of each gene.

Gene	Upstream primer	Downstream primer
β-actin	5′-CATGTACGTTGCTATCCAGGC-3′	5′-CTCCTTAATGTCACGCACGAT-3′
A2AR	5′-TGGCTTGGTGACGGGTATG-3′	5′-CGCAGGTCTTTGTGGAGTTC-3′
miR-214	5′-GCGCGTGAGCAGGCTGGAGA-3′	5′-CACAGCAGGCACAGAC-3′
TGF-β	5′-GAAGTGCATCCACGAGCCAA-3′	5′-GCTGCACTTGCAGGACGCGC-3′
U6	5′- AAAGCAAATCATCGCACGAC -3′	5′- GTACAACACATTGTTTCCTCC-3′

### Protein expression detected by WB

The scar tissue and normal human skin tissues, as well as HSF and various groups of KFBs, were washed three times with PBS containing the proteinase inhibitor phenylmethylsulfonyl fluoride (PMSF). Then, 200 μL of pre-prepared cell lysis buffer Radio Immunoprecipitation Assay (RIPA) + PMSF (RIPA: PMSF = 1:100) was applied, and the mixture was incubated on ice for 30 min at 4 °C and centrifuged at 12,000 rpm for 10 min to collect the supernatant containing total protein. The extracted total protein was quantified adopting bicinchoninic acid (BCA) assay for protein concentration measurement. Cellular proteins were separated using sodium dodecyl sulfate polyacrylamide gel electrophoresis, and then transferred onto polyvinylidene fluoride membranes using a wet transfer methodology. Membranes were blocked with 5% skim milk. Primary antibodies were applied at a 1:1000 dilution and incubated overnight at 4 °C. Membranes were washed thrice with TBST for 10 min each, and incubated with HRP-conjugated secondary antibodies at 25 °C for 2 h. The membranes were washed thrice with TBST for 10 min each, and then incubated with a chromogenic substrate. The protein level was analyzed by measuring the grayscale value using an imaging system.

### Cell *Pro* activity detected by CCK8

At 70–80% confluency, cells were collected and resuspended. The cell suspension was prepared for each cell type at 5 × 10^3^ cells per well in a volume of 200 μL in a 96-well plate with 5 replicates per condition. The plate was incubated in a cell culture incubator for 24, 48, and 72 h. Medium was discarded from each well after incubation, and 100 μL CCK-8 reagent mixed with DMEM (1:10) was applied. The plate was incubated for an additional 3 h. Absorbance at 450 nm was measured using a microplate reader, and data were analyzed.

### Flow cytometry (FCM)

After 48 h of transfection and culture, cells were digested and collected. After cells were washed with pre-chilled PBS, a single-cell suspension was prepared with binding buffer at 1–5 × 10^6^ cells/mL. 100 μL cell suspension was mixed with pre-chilled 70% ethanol and fixed for 1–2 h. After centrifugation, cells were resuspended in 3 mL PBS for 5 min, and then centrifuged again. 5 μL Annexin V-FITC was applied in the cell pellet, mixed thoroughly, and incubated for 15 min in the dark. Then, 5 μL propidium iodide (PI) staining solution was applied for staining for 30 min in the dark at 4 ℃. The apoptotic status of the cells was detected by FCM.

### Statistical analysis

Statistical analysis was conducted using SPSS 19.0, with quantitative data presented as mean ± standard deviation (${\overline{\text{x}}}$  ± s). Analysis of Variance (ANOVA) was employed for the comparison of quantitative data among multiple groups, and t-tests were utilized for the analysis of metric data between two groups. A significance level of *P* < 0.05 was considered statistically significant.

### Ethical statement

The content of this study has been approved by the Medical Ethics Committee of the Second People’s Hospital of Wuxi City. All methods were carried out in accordance with relevant guidelines and regulations. Both the research patients themselves and their family members had been informed about the relevant details of the study. Informed consent is obtained from all participants. They were aware of the study’s content and have provided written consent to participate in the research.

## Results

### FTIR results of MC-NPs

**Figure 1:**
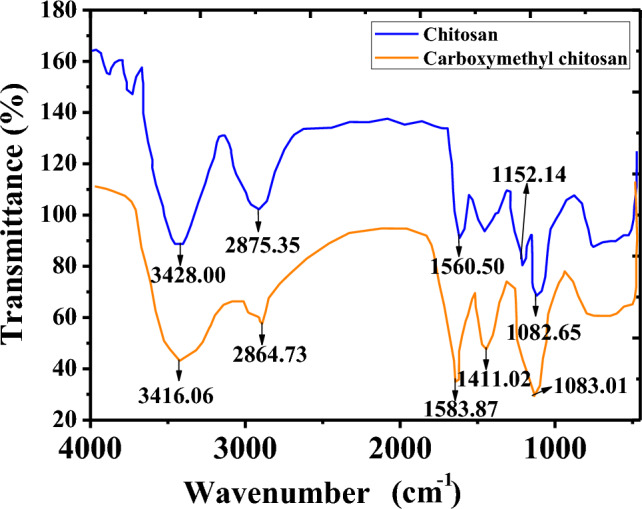
FTIR Spectra of Chitosan and MC-NPs.

Before conducting the FTIR analysis of MC-NPs, separate FTIR analyses were performed on chitosan and carboxymethyl chitosan separately. The results, illustrated in Fig. 1, were analyzed, revealing differences in characteristic absorption peaks were observed between chitosan and carboxymethyl chitosan. Carboxymethyl chitosan exhibited a characteristic absorption peak of the -COOH functional group at 1583.87/cm and a characteristic absorption peak of the –C–O– functional group at 1083.01/cm, indicating the successful preparation of carboxymethyl chitosan.

**Figure 2:**
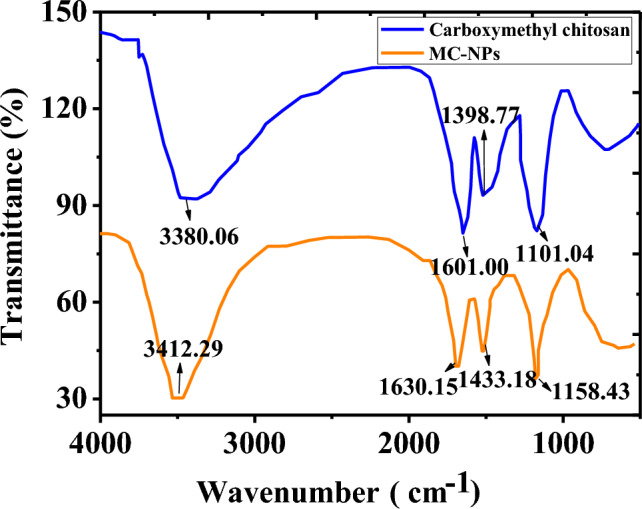
FTIR Spectra of carboxymethyl chitosan and MC-NPs.

The FTIR results of carboxymethyl chitosan and MC-NPs were presented in Fig. 2. The characteristic absorption peak of the protonated –COOH group was observed around 1740/cm, whereas the deprotonated -COOH group exhibited peaks around 1600/cm and 1410/cm. Carboxymethyl chitosan displayed prominent characteristic absorption peaks at 1601.00/cm and 1398.77/cm, indicating a significant anionic nature. In MC-NPs, the absorption peak of the protonated –COOH group has significantly shifted compared to carboxymethyl chitosan, appearing prominently at 1630.15/cm and 1433.18/cm. This shift suggested that in MC-NPs, the -COOH groups of carboxymethyl chitosan had undergone ion crosslinking with Ca2+. Both carboxymethyl chitosan and MC-NPs exhibited distinct characteristic absorption peaks of amino groups at 3412/cm, indicating that the amino groups of carboxymethyl chitosan did not participate in ion reactions.

### The morphological characteristics and particle size distribution of MC-NPs

**Figure 3:**
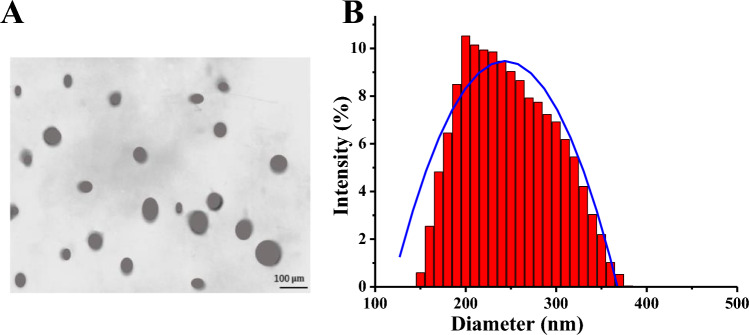
FTIR Spectra of carboxymethyl chitosan and MC-NPs. (**A**: TEM image of MC-NPs; **B**: Particle size distribution of MC-NPs).

The morphological characteristics and particle size distribution of MC-NPs were summarized in Fig. 3. The particles of MC-NPs exhibited a regular spherical shape, and the particle size distribution was relatively uniform. The average particle size of MC-NPs was 236.47 ± 4.98 nm.

### Influences of MC-NPs on proliferation ability of KFBs

**Figure 4:**
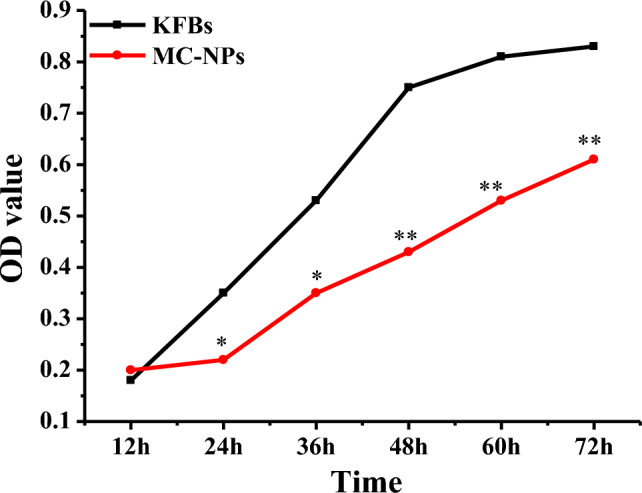
Influences of MC-NPs on proliferation ability of KFBs. (*Indicated a statistically significant difference compared to the control KFBs, *P* < 0.05; ** indicated a significant difference compared to the control KFBs, *P* < 0.01).

The statistical results of the impact of MC-NPs on the proliferation ability of KFBs were presented in Fig. 4. As the cultivation time increased, both the control group and the MC-NPs group of KFBs exhibited a significant upward trend in OD values. Moreover, within the same time frame, the cell OD values of the MC-NPs group were lower than those of KFBs.

### Analysis of miR-214 and A2AR in human KFBs and tissues

**Figure 5:**
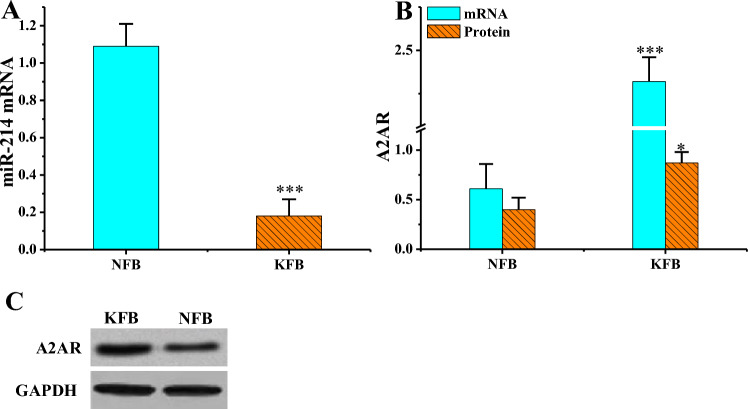
MiR-214 and A2AR levels in different cells (n = 3). (*P < 0.05, ***P < 0.001 vs. NFB). (**A**: miR-214 mRNA level; **B**: A2AR mRNA and protein levels; **C**: A2AR WB print).

The levels of miR-214 and A2AR in KFB and NFB were detected by RT-PCR and WB, respectively (Fig. 5). The mRNA of miR-214 in KFB was drastically inferior to that in NFB (*P* < 0.001), while A2AR mRNA and protein levels in KFB were greatly superior to those in NFB, with considerable differences in mRNA levels (*P* < 0.001) and substantial differences in protein levels (*P* < 0.05) in pairwise comparisons.

**Figure 6:**
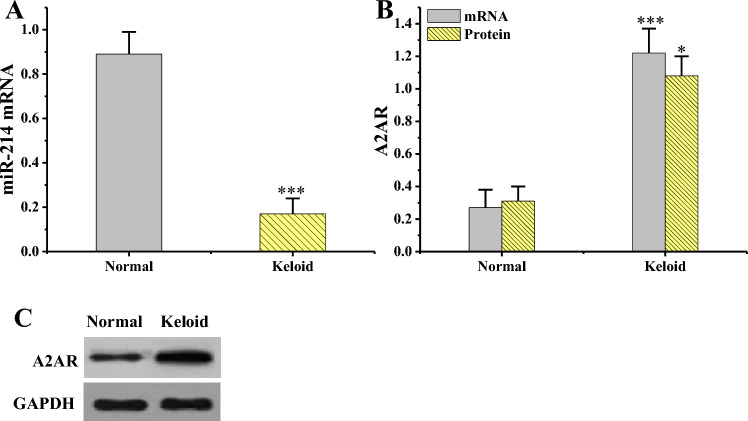
MiR-214 and A2ARs in various tissues (n = 3). (*P < 0.05, ***P < 0.001 vs. normal skin tissue.). (**A**: miR-214 mRNA level; **B**: A2AR mRNA and protein levels; **C**: A2AR WB print).

Using both RT-PCR and WB methods, miR-214 and A2AR in keloid tissues and normal skin tissues were detected (Fig. 6). MiR-214 mRNA level in keloid tissues was markedly inferior to that in normal skin tissues (*P* < 0.001), while A2AR mRNA and protein levels in keloid tissues were obviously superior to those in normal skin tissues. The mRNA comparison between groups showed extremely notable differences (*P* < 0.001), and protein comparison showed great differences (*P* < 0.05).

### Effect of miR-214 on *Pro* and *Apo* of KFBs

**Figure 7:**
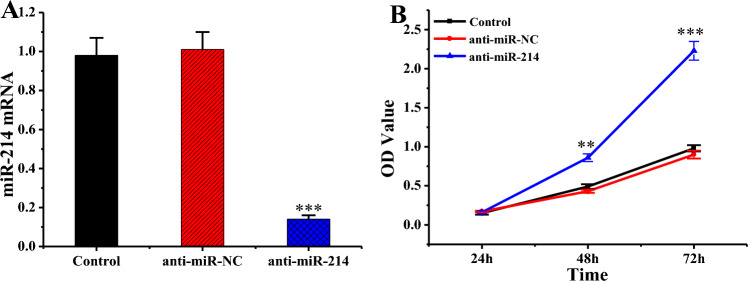
MiR-214 levels and cell *Pro* among various groups (n = 3). (**P < 0.001, *** P < 0.001 vs. anti-miR-NC.). (**A**: mRNA levels of miR-214 in various groups; **B**: comparison of cell OD values in various groups and at various time periods).

RT-qPCR revealed that miR-214 was substantially decreased in anti-miR-214 group cells compared to Ctrl and anti-miR-NC groups (*P* < 0.001) (Fig. 7A). The impact of miR-214 on the *Pro* of KFBs was detected using the CCK-8 method (Fig. 7B). With the extension of culture time, the OD values of KFB cells in various groups showed a drastic upward trend. At 24 h, the OD values demonstrated inconsiderable differences among Ctrl, anti-miR-NC, and anti-miR-214 groups (*P* > 0.05); at 48 h, the OD value of the anti-miR-214 group was remarkably superior to that of Ctrl and anti-miR-NC groups (*P* < 0.01); at 72 h, the OD value of the anti-miR-214 group increased notably versus Ctrl and anti-miR-NC groups (*P* < 0.001).

**Figure 8:**
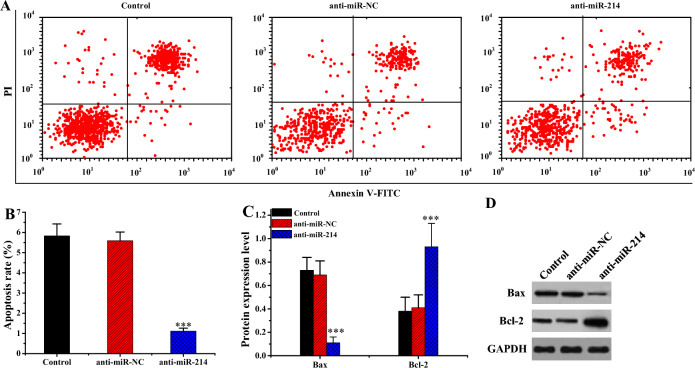
Influence of miR-214 on the apoptotic capability of KFBs (n = 3). (***P < 0.001 vs. Ctrl or anti-miR-NC). (**A**: FCM chart; **B**: comparison of *Apo* rates; **C**: comparison of levels of apoptotic proteins; **D**: apoptotic WB print).

The results of FCM revealed that *Apo* rate of cells in anti-miR-214 group was remarkably lower than Ctrl and anti-miR-NC groups (*P* < 0.001) (Fig. 8A and B). WB was used to detect the influence of miR-214 on the levels of *Apo*-related genes in hypertrophic scar fibroblasts. The results are illustrated in Fig. 8C and D). Relative to Ctrl and anti-miR-NC groups, the anti-miR-214 group showed a notable decrease in the protein level of *Apo* gene Bax (*P* < 0.001) and a major increase in protein level of anti-apoptotic gene Bcl-2 (*P* < 0.001).

### TGF-β levels in various groups of cells

**Figure 9:**
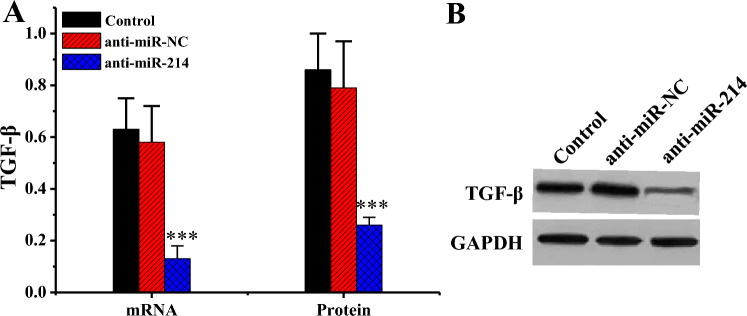
TGF-β levels in various groups of cells (n = 3). (***P < 0.001 vs. Ctrl or anti-miR-NC). (**A**: comparison of TGF-β mRNA levels; **B**: TGF-β WB print).

RT-qPCR results revealed that mRNA levels of TGF-β gene in the anti-miR-214 group were substantially inferior to Ctrl and the anti-miR-NC groups (*P* < 0.001). WB revealed that relative to the protein level of TGF-β in the anti-miR-214 group was substantially decreased versus Ctrl and anti-miR-NC groups (*P* < 0.001) (Fig. 9).

### MiR-214 target negative regulation A2AR

**Figure 10:**
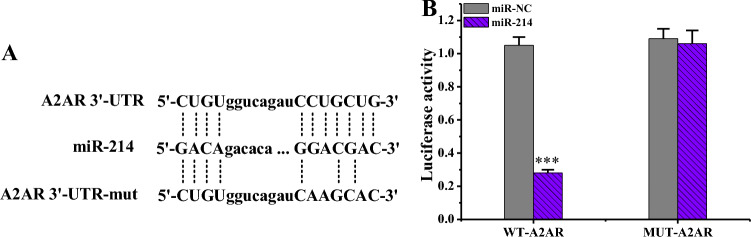
Analysis of targeting relationship between miR-214 and A2AR (n = 3). (***P < 0.001 vs. cells transfected with miR-NC and WT-A2AR). (**A**: targeting nucleotide sequence between miR-214 and A2AR; **B**: comparison of luciferase activities among various groups).

According to bioinformatics target prediction results, continuous complementary binding sites existed between miR-214 and A2AR (Fig. 10A). Further validation of the targeting relationship between miR-214 and A2AR was performed adopting a dual luciferase assay (Fig. 10B). The luciferase activity in KFB cells co-transfected with miR-214 mimics and WT-A2AR was considerably inferior to those transfected with miR-NC and WT-A2AR (*P* < 0.001). In addition, the luciferase activity change in KFB cells co-transfected with miR-214 mimics and MUT-A2AR was not markedly different compared to cells co-transfected with miR-NC and MUT-A2AR (*P* > 0.05).

**Figure 11:**
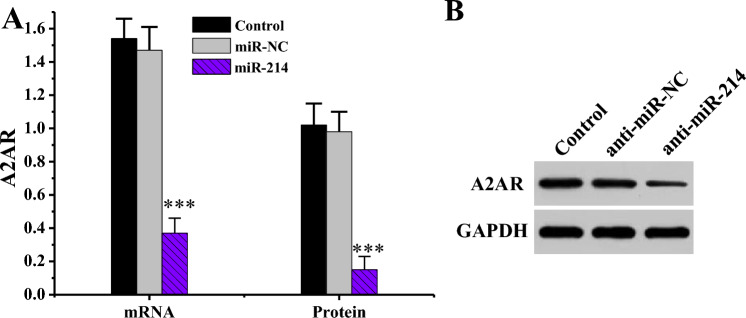
Impact of miR-214 on A2AR gene level (n = 3). (***P < 0.001 vs. Ctrl or miR-NC). (**A**: comparison of mRNA levels of A2AR gene; **B**: A2AR WB print).

How miR-214 impacted A2AR gene were demonstrated by RT-qPCR and WB (Fig. 11), and the outcome indicated that A2AR mRNA and protein levels were substantially decreased in miR-214 mimics group relative to Ctrl and miR-NC groups (*P* < 0.001).

### Influence of interfering A2AR on *Pro* and *Apo* of KFBs

The statistical results of the effects of interfering with A2AR on the *Pro* and *Apo* ability of hypertrophic scar fibroblasts are illustrated in Table 2. A2AR gene mRNA and protein levels in si-A2AR group were substantially decreased versus Ctrl and the si-con groups (*P* < 0.001), and the OD values detected by cell viability assay were substantially decreased (*P* < 0.001), but *Apo* rate was dramatically enhanced (*P* < 0.001).

**Table 2:** Effects of interference with A2AR on *Pro* and *Apo* of KFBs (n = 3).

Group	A2AR mRNA level	A2AR protein level	Cell viability (OD value)	*Apo* rate (%)
Ctrl	1.54 ± 0.23	0.81 ± 0.15	1.01 ± 0.14	4.52 ± 0.98
si-con	1.50 ± 0.21	0.79 ± 0.16	0.97 ± 0.13	4.46 ± 0.87
si-A2AR	0.28 ± 0.16***	0.12 ± 0.09***	0.17 ± 0.08***	11.38 ± 1.74***
*F*	85.216	60.031	104.194	19.216
*P*	< 0.001	< 0.001	< 0.001	< 0.001

***P < 0.001 vs. Ctrl or si-con.

### Interference with A2AR reversed the effect of miR-214 inhibition on KFBs

The statistical results of the reversal of A2AR interference on the inhibition of miR-214 in hypertrophic scar fibroblasts are illustrated in Table 3. OD of cell viability detection in the anti-miR-214 group was dramatically increased relative to anti-miR-NC group (*P* < 0.001), while the *Apo* rate was substantially decreased (*P* < 0.001). Furthermore, OD of cell viability detection in anti-miR-214 + si-A2AR group was substantially decreased versus that of anti-miR-214 + si-con group (*P* < 0.001), while *Apo* rate was dramatically enhanced (*P* < 0.001).

**Table 3:** Disturbance of A2AR reverses the effect of inhibition of miR-214 on KFBs (n = 3).

Group	Cell viability (OD value)	*Apo* rate (%)
anti-miR-NC	0.96 ± 0.14	6.14 ± 0.93
anti-miR-214	2.63 ± 0.25***	1.09 ± 0.29***
anti-miR-214 + si-con	2.58 ± 0.26	1.25 ± 0.31
anti-miR-214 + si-A2AR	0.43 ± 0.09***	4.98 ± 1.03***
*F*	127.572	32.587
*P*	< 0.001	< 0.001

***P < 0.001 vs. anti-miR-NC or anti-miR-214 + si-con.

### Effect of interfering A2AR on TGF-β level in KFBs

**Figure 12:**
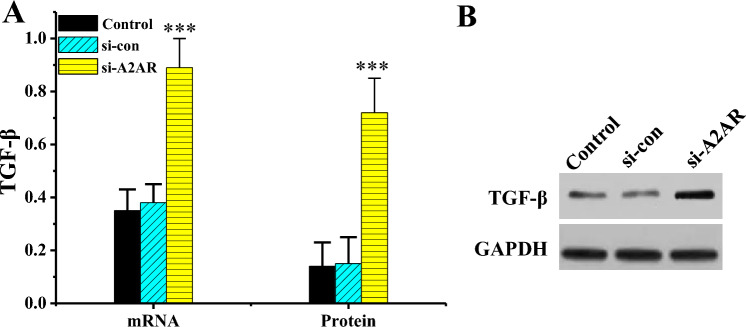
TGF-β levels in various groups of cells (n = 3). (***P < 0.001 vs. Ctrl or si-con). (**A**: comparison of TGF-β mRNA levels; **B**: TGF-β WB print).

RT-qPCR results revealed that TGF-β mRNA and protein levels in si-A2AR group were dramatically increased versus Ctrl and si-con groups (*P* < 0.001). WB detection results revealed that in contrast to Ctrl and anti-miR-NC groups, TGF-β protein in si-A2AR group was dramatically increased (*P* < 0.001) (Fig. 12).

## Discussion

Scar formation involves an increase in connective tissue, with excessive *Pro* of scar fibroblasts and abnormal cell *Apo* being the main contributors to this process^23^. In this work, MC-NPs were prepared based on carboxymethyl chitosan, and the results demonstrated that the particles of MC-NPs exhibited a regular spherical shape with an average particle size of 236.47 ± 4.98 nm. The uniform spherical morphology indicates a certain degree of control in the preparation process of MC-NPs, which is crucial for potential biomedical applications. Spherical particles offer better particle dispersion, stability, and size consistency^24^. The particle size plays a crucial role in determining the properties and applications of MC-NPs. Smaller particle sizes can enhance the biodegradability, penetration ability, and particle stability, making them favorable for applications such as drug delivery and gene delivery^25^. Simultaneously, we observed that within the same time frame, the cell OD values of the MC-NPs group were lower than those of KFBs. This suggests a potential inhibitory effect of MC-NPs on cell viability and proliferation. This inhibitory effect could be attributed to the characteristics and interaction mechanisms of MC-NPs. The affinity between cells and carboxymethyl chitosan nanoparticles may not be strong, leading to inadequate cell-nanoparticle contact and consequently reducing cellular metabolic activity^26^. It is possible that MC-NPs might interfere with normal cellular functions or cell signaling pathways, thus diminishing cellular metabolic activity^27^.

Multiple research results suggest that miRNAs play a crucial role in scar formation and represent potential targets for scar treatment^28,29^. MiRNAs can slow down scar formation by inhibiting the target gene expression and activating or inhibiting related pathways^30^. MiR-214 acts in cell growth, *Pro*, and *Apo*. Studies have indicated that the level of miR-214 is down-regulated in lung adenocarcinoma, and interference with miR-214 causes reduced *Pro* ability of lung adenocarcinoma cells and promotes cell *Apo*, suggesting that miR-214 can promote the *Pro* of lung adenocarcinoma cells while also possessing an anti-apoptotic effect^31^. Additionally, miR-214 has been found to promote the *Pro* and invasion of T-cell lymphoblastic lymphoma cells while inhibiting their *Apo* by targeting and regulating the level of the AIFM2 protein^32^. He et al.^33^ found that miR-214 participates in the migration and invasion of liver cancer cells by regulating its target gene level. Zhang et al.^34^ reported that miR-214 is involved in survival of osteoblasts and extracellular matrix formation by regulating the level of type IV collagen. Currently, research on changes in miR-214 levels is mainly focused on tumor development. Our results revealed that miR-214 levels in keloid tissues and KFB were significantly lower compared to NFB and normal skin tissues (*P* < 0.001). The decreased miR-214 may be involved in the formation of keloids. At 72 h, the OD value of cells in the anti-miR-214 group was increased versus Ctrl and anti-miR-NC groups (*P* < 0.001), and the *Apo* rate was notably reduced (*P* < 0.001). *Apo* gene Bax protein level was substantially decreased (*P* < 0.001), while the protein level of the anti-*Apo* gene Bcl-2 was dramatically increased (*P* < 0.001). Thus, inhibiting miR-214 can promote the *Pro* of KFBs cells, inhibit cell *Apo*, increase the anti-apoptotic protein Bcl-2 level, and decrease the pro-apoptotic protein Bax.

To investigate the molecular mechanism of miR-214 in the formation of hypertrophic scars, bioinformatics methodologies were adopted to predict target genes of miR-214, and A2AR emerged as one of them. MiR-214 was found to negatively regulated the level of A2AR. Adenosine is dramatically increased in cells or tissues damaged by ischemia, inflammatory reactions, and other injuries, and A2AR may participate in this transduction process^35^. Adenosine receptor activation has a potential pro-angiogenic effect, which is associated with the downregulation of thrombospondin-1 adhesive protein TAP1 by A2AR agonists^36^. The results indicated that both mRNA and protein of A2AR in KFB were superior to those in NFB, and the mRNA levels had extremely notable differences (*P* < 0.001) when compared pairwise. The protein levels had statistical differences (*P* < 0.05) when compared pairwise. Additionally, the si-A2AR group exhibited significantly reduced mRNA and protein levels of the A2AR gene compared to the Ctrl and si-con groups (*P* < 0.001). This suggests that A2AR level is increased in KFBs or tissues. Low A2AR level can lead to a decrease in number of microvessels during wound healing, which in turn reduces peritoneal fibrosis^37^. TGF-β is a multi-biological effect cytokine that acts in embryonic development and tissue damage repair and in diseases such as wound healing, scar tissue *Pro*, and fibrosis^38^. Scar formation is greatly correlated with TGF-β level. TGF-β can promote DNA synthesis and collagen synthesis in both scar tissue and NFBs^39^. Proliferative scar fibroblasts exhibit a significant increase in TGF-β levels, enhancing the chemotactic ability of smooth muscle cells and fibroblasts, leading to the aggregation of fibrotic cells in extracellular matrix, ultimately resulting in scar formation^40^. Furthermore, the mRNA and protein levels of the TGF-β gene in anti-miR-214 group were substantially decreased compared to Ctrl and anti-miR-NC groups (*P* < 0.001). In contrast, relative to Ctrl and si-con groups, TGF-β gene mRNA and protein levels were dramatically enhanced in the si-A2AR group (*P* < 0.001). Hence, interfering with A2AR or miR-214 can change the level of TGF-β in scar fibroblasts, indicating that miR-214 participates in scar formation by targeting the regulation of A2AR levels to change the level of TGF-β.

## Conclusion

In this work, the ion cross-linking was employed to prepare MC-NPs and analyze the impact of MC-NPs on KFBs proliferative capability. Simultaneously, the work investigated the mechanistic roles of miR-214 and adenosine A2AR in fibroblasts within hypertrophic scars. The results indicated that MC-NPs exerted a certain inhibitory effect on KFBs. MiR-214, through targeted regulation of A2AR expression levels, promoted the proliferation of fibroblasts within hypertrophic scars and inhibited their apoptosis, ultimately contributing to the occurrence of hypertrophic scars. The limitations of this work include the lack of clarification regarding the specific mechanism of MC-NPs on KFBs and the unclear involvement of miR-214 and A2AR in the regulation of the TGF-β/mad signaling pathway. In future work, further analysis based on animal experiments will be conducted to explore the correlation between miR-214, A2AR, and the TGF-β/mad signaling pathway. In conclusion, the experimental results of this work offered a theoretical foundation and guidance for understanding the mechanism and potential treatments of hypertrophic scars.

### Supplementary Information

Supplementary Information.

## Data Availability

All data generated or analysed during this study are included in this published article [and its supplementary information files].

## Abbreviations

A2AR: A2A receptor
KFB: Keloid fibroblast
NFB: Normal fibroblast
CCK8: Cell Counting Kit-8
FCM: Flow cytometry
Apo: Apoptosis
WB: Western blotting
Pro: Proliferation
FBS: Fetal bovine serum
DMEM: Dulbecco’s Modified Eagle Medium
PBS: Phosphate-buffered saline
HRP: Horseradish peroxidase
PMSF: Phenylmethylsulfonyl fluoride
RIPA: Radio Immunoprecipitation Assay
BCA: Bicinchoninic acid
PI: Propidium iodide
MC-NPs: Carboxymethyl chitosan nanoparticles

## References

[1] Ogawa R, Dohi T, Tosa M, Aoki M, Akaishi S (2021). The latest strategy for keloid and hypertrophic scar prevention and treatment: The nippon medical school (NMS) protocol. J. Nippon Med. Sch..

[2] Wang ZC, Zhao WY, Cao Y, Liu YQ, Sun Q, Shi P, Cai JQ, Shen XZ, Tan WQ (2020). The roles of inflammation in keloid and hypertrophic scars. Front. Immunol..

[3] Direder M, Weiss T, Copic D, Vorstandlechner V, Laggner M, Pfisterer K, Mildner CS, Klas K, Bormann D, Haslik W, Radtke C, Farlik M, Shaw L, Golabi B, Tschachler E, Hoetzenecker K, Ankersmit HJ, Mildner M (2022). Schwann cells contribute to keloid formation. Matrix Biol..

[4] Ogawa R (2017). Keloid and hypertrophic scars are the result of chronic inflammation in the reticular dermis. Int. J. Mol. Sci..

[5] Shim J, Oh SJ, Yeo E, Park JH, Bae JH, Kim SH, Lee D, Lee JH (2022). Integrated analysis of single-cell and spatial transcriptomics in keloids: Highlights on fibrovascular interactions in keloid pathogenesis. J. Invest. Dermatol..

[6] Liu S, Yang H, Song J, Zhang Y, Abualhssain ATH, Yang B (2022). Keloid: Genetic susceptibility and contributions of genetics and epigenetics to its pathogenesis. Exp. Dermatol..

[7] Elsaie ML (2021). Update on management of keloid and hypertrophic scars: A systemic review. J. Cosmet. Dermatol..

[8] Mousazadeh H, Pilehvar-Soltanahmadi Y, Dadashpour M, Zarghami N (2021). Cyclodextrin based natural nanostructured carbohydrate polymers as effective non-viral siRNA delivery systems for cancer gene therapy. J. Control Release..

[9] Salmani Javan E, Lotfi F, Jafari-Gharabaghlou D, Mousazadeh H, Dadashpour M, Zarghami N (2022). Development of a magnetic nanostructure for co-delivery of metformin and silibinin on growth of lung cancer cells: Possible action through leptin gene and its receptor regulation. Asian Pac. J. Cancer Prev..

[10] Arya SS, Rookes JE, Cahill DM, Lenka SK (2022). Chitosan nanoparticles and their combination with methyl jasmonate for the elicitation of phenolics and flavonoids in plant cell suspension cultures. Int. J. Biol. Macromol..

[11] Schlachet I, Trousil J, Rak D (2019). Chitosan-graft-poly(methyl methacrylate) amphiphilic nanoparticles: Self-association and physicochemical characterization. Carbohydr. Polym..

[12] Talaei F, Azhdarzadeh M, Hashemi Nasel H (2011). Core shell methyl methacrylate chitosan nanoparticles: In vitro mucoadhesion and complement activation. Daru.

[13] Yang JI, Lee HL, Yun JJ (2022). pH and redox-dual sensitive chitosan nanoparticles having methyl ester and disulfide linkages for drug targeting against cholangiocarcinoma cells. Mater. (Basel).

[14] Nejati K, Rastegar M, Fathi F, Dadashpour M, Arabzadeh A (2022). Nanoparticle-based drug delivery systems to overcome gastric cancer drug resistance. J. Drug Deliv. Sci. Technol..

[15] Macarak EJ, Wermuth PJ, Rosenbloom J, Uitto J (2021). Keloid disorder: Fibroblast differentiation and gene expression profile in fibrotic skin diseases. Exp. Dermatol..

[16] Sun C, Wang B, Hao S (2022). Adenosine-A2A receptor pathway in cancer immunotherapy. Front. Immunol..

[17] Zheng X, Wang D (2020). The adenosine A2A receptor agonist accelerates bone healing and adjusts Treg/Th17 cell balance through interleukin 6. Biomed. Res. Int..

[18] Guerrero A (2018). A2A adenosine receptor agonists and their potential therapeutic applications. An update. Curr. Med. Chem..

[19] Zhong L, Bian L, Lyu J, Jin H, Liu Z, Lyu L, Lu D (2018). Identification and integrated analysis of microRNA expression profiles in keloid. J. Cosmet. Dermatol..

[20] Xu M, Sun J, Yu Y, Pang Q, Lin X, Barakat M, Lei R, Xu J (2020). TM4SF1 involves in miR-1–3p/miR-214–5p-mediated inhibition of the migration and proliferation in keloid by regulating AKT/ERK signaling. Life Sci..

[21] Haghani-Dogahe Z, Hadadi R, Esmailzadeh M, Mobayen M (2023). Comparing intralesional triamcinolone and verapamil-triamcinolone injections in keloids: A single-blinded randomised clinical trial. Int. Wound J..

[22] Migone C, Mattii L, Giannasi M (2020). Nanoparticles based on quaternary ammonium chitosan-methyl-β-cyclodextrin conjugate for the neuropeptide dalargin delivery to the central nervous system: An in vitro study. Pharmaceutics.

[23] Wang Q, Wang P, Qin Z, Yang X, Pan B, Nie F, Bi H (2021). Altered glucose metabolism and cell function in keloid fibroblasts under hypoxia. Redox Biol..

[24] Zielińska A, Carreiró F, Oliveira AM (2020). Polymeric nanoparticles: Production, characterization, toxicology and ecotoxicology. Molecules.

[25] Li X, Montague EC, Pollinzi A, Lofts A, Hoare T (2022). Design of smart size-, surface-, and shape-switching nanoparticles to improve therapeutic efficacy. Small.

[26] Schlachet I, Moshe-Halamish H, Sosnik A (2020). Mixed amphiphilic polymeric nanoparticles of chitosan, poly(vinyl alcohol) and poly(methyl methacrylate) for intranasal drug delivery: A preliminary in vivo study. Molecules.

[27] Piras AM, Fabiano A, Chiellini F, Zambito Y (2018). Methyl-β-cyclodextrin quaternary ammonium chitosan conjugate: Nanoparticles vs macromolecular soluble complex. Int. J. Nanomed..

[28] Hietanen KE, Järvinen TA, Huhtala H, Tolonen TT, Kuokkanen HO, Kaartinen IS (2019). Treatment of keloid scars with intralesional triamcinolone and 5-fluorouracil injections—a randomized controlled trial. J. Plast. Reconstr. Aesthet. Surg..

[29] He Y, Zhang Z, Yin B, Li S, Wang P, Lan J, Lian W, Jia C (2022). Identifying miRNAs associated with the progression of keloid through mRNA-miRNA network analysis and validating the targets of miR-29a-3p in keloid fibroblasts. Biomed. Res. Int..

[30] Lyu L, Zhao Y, Lu H, Liu Z, Guo J, Lu D, Li X (2019). Integrated interaction network of MicroRNA target genes in keloid scarring. Mol. Diagn. Ther..

[31] Zhang J, Wu J (2021). The potential roles of exosomal miR-214 in bone metastasis of lung adenocarcinoma. Front. Oncol..

[32] Fan FY, Deng R, Yi H, Sun HP, Zeng Y, He GC, Su Y (2017). The inhibitory effect of MEG3/miR-214/AIFM2 axis on the growth of T-cell lymphoblastic lymphoma. Int. J. Oncol..

[33] He GN, Bao NR, Wang S, Xi M, Zhang TH, Chen FS (2021). Ketamine induces ferroptosis of liver cancer cells by targeting lncRNA PVT1/miR-214-3p/GPX4. Drug Des. Dev. Ther..

[34] Zhang K, Liu X, Tang Y, Liu Z, Yi Q, Wang L, Geng B, Xia Y (2022). Fluid shear stress promotes osteoblast proliferation and suppresses mitochondrial-mediated osteoblast apoptosis through the miR-214-3p-ATF4 signaling axis. Physiol. Res..

[35] Bonyanian Z, Walker M, Du-Toit E, Rose'Meyer RB (2019). Multiple adenosine receptor subtypes stimulate wound healing in human EA.hy926 endothelial cells. Purinergic Signal..

[36] Zhu W, Dong Y, Xu P, Pan Q, Jia K, Jin P, Zhou M, Xu Y, Guo R, Cheng B (2022). A composite hydrogel containing resveratrol-laden nanoparticles and platelet-derived extracellular vesicles promotes wound healing in diabetic mice. Acta Biomater..

[37] Galeano M, Pallio G, Irrera N, Mannino F, Bitto A, Altavilla D, Vaccaro M, Squadrito G, Arcoraci V, Colonna MR, Lauro R, Squadrito F (2021). Polydeoxyribonucleotide: A promising biological platform to accelerate impaired skin wound healing. Pharmaceut. (Basel).

[38] Zhang T, Wang XF, Wang ZC, Lou D, Fang QQ, Hu YY, Zhao WY, Zhang LY, Wu LH, Tan WQ (2020). Current potential therapeutic strategies targeting the TGF-β/Smad signaling pathway to attenuate keloid and hypertrophic scar formation. Biomed. Pharmacother..

[39] Lei R, Li J, Liu F, Li W, Zhang S, Wang Y, Chu X, Xu J (2019). HIF-1α promotes the keloid development through the activation of TGF-β/Smad and TLR4/MyD88/NF-κB pathways. Cell Cycle..

[40] Liu X, Chen W, Zeng Q, Ma B, Li Z, Meng T, Chen J, Yu N, Zhou Z, Long X (2022). Single-cell RNA-sequencing reveals lineage-specific regulatory changes of fibroblasts and vascular endothelial cells in keloids. J. Invest. Dermatol..

